# Two-Way Affective Modeling for Hidden Movie Highlights’ Extraction

**DOI:** 10.3390/s18124241

**Published:** 2018-12-03

**Authors:** Zheng Wang, Xinyu Yan, Wei Jiang, Meijun Sun

**Affiliations:** 1 Division of Intelligence and Computing, Tianjin University, Tianjin 300072, China; xinyuyan@tju.edu.cn (X.Y.); jiangweitju@163.com (W.J.); sunmeijun@tju.edu.cn (M.S.); 2 School of Computer Information and Engineering, Changzhou Institute of Technology, Changzhou 213032, China

**Keywords:** affective computing, movie exciting degree, excitement modeling, movie highlights’ extraction

## Abstract

Movie highlights are composed of video segments that induce a steady increase of the audience’s excitement. Automatic movie highlights’ extraction plays an important role in content analysis, ranking, indexing, and trailer production. To address this challenging problem, previous work suggested a direct mapping from low-level features to high-level perceptual categories. However, they only considered the highlight as intense scenes, like fighting, shooting, and explosions. Many hidden highlights are ignored because their low-level features’ values are too low. Driven by cognitive psychology analysis, combined top-down and bottom-up processing is utilized to derive the proposed two-way excitement model. Under the criteria of global sensitivity and local abnormality, middle-level features are extracted in excitement modeling to bridge the gap between the feature space and the high-level perceptual space. To validate the proposed approach, a group of well-known movies covering several typical types is employed. Quantitative assessment using the determined excitement levels has indicated that the proposed method produces promising results in movie highlights’ extraction, even if the response in the low-level audio-visual feature space is low.

## 1. Introduction

Human–computer interactions (HCI) are crucial for user-friendly interactions between human users and computer systems. HCI is not only requested to provide effective input/output, it is also expected to understand the intentions of users and the environment for better service-oriented interactions. These have raised new challenges beyond conventional multimodal HCI, including audio, images, video, and graphics [1,2,3]. Recently, thanks to emerging sensors and sensing techniques, HCI has been further developed for immersive and affective communication between human users and computer systems. Examples can be found in virtual reality-based experiences, electroencephalogram-enabled brain–computer interfaces, and smart interactions between humans and robots. In this paper, we propose a new method to understand highlight affective segments in movies, which is useful for further sensor and HCI techniques. The proposed technology used to develop such a sensor is automatic video content analysis, which aims to reveal both the objective entities and hidden subjective feelings or emotions from movies. It overcomes the problems caused by the explosively-increasing repository of online movies. It can select movies more easily from a large video database than a typical viewer could.

The concerned research problems and used methodologies cross several technical fields, ranging from cinematographic [4], cognitive psychology [5,6] to video content analysis.

In the last two decades, video content analysis has attracted considerable attention, and relevant approaches can be divided into two categories. The first is objective content analysis, which aims at extracting “fact”-based information, including content extraction [7,8,9], audio-visual affective extraction [10,11,12,13,14], event detection [15,16], etc. The second is affective video analysis, where feelings or emotions within the clip are analyzed [17,18]. In some contexts, an efficient method is needed to determine the more or less exciting parts of a movie. This is called video highlights’ analysis, which also belongs to the second category [19].

Most relevant work in video analysis is based on realistic imaging modalities, represented by photographs along time and related events of (real-world) objects [16]. There exists a rough correspondence between objects/events and video content. Generally, low-level audio-visual features in videos are first extracted, which are then employed to detect and understand the objects and/or events on the basis of a certain ground truth database [20,21,22,23,24].

For affective video analysis [25,26,27,28,29,30], however, this process is quite different, mainly because the feelings and emotions involved are abstractive high-level semantics. Due to the difficulty in defining objective models to describe the subjective affective value, relevant research is rare and recent. Early work was based on a bottom-up method, which relies on a direct mapping from low-level features to affective semantic meanings. In Reference [31], a 2D valence-arousal emotional space was presented for affective video content analysis. In Wang and Cheong [32], fusion of audio and visual features followed by a support vector machine (SVM) inference engine were proposed for affective information extraction. The combination of psychology and cinematography knowledge for affective video analysis can be found in References [20,32,33,34].

Due to the relatively simple structure and clear semantics contained, highlights’ extraction from sports, music, and news videos have been intensively studied [19,35,36,37]. Existing work mainly focused on event-based approaches, where modeling from features to events is required [35,38]. However, work on highlights’ extraction from generic videos under more complex structures and contents, such as movies, is still rare and limited. In Reference [39] of Xu et al., a hierarchical structure for emotion categories by using arousal- and valence-related features was introduced, which shows better performance for action and horror films than for drama and comedy. In Reference [3], an affective framework was proposed from audiovisual and film grammar features to movie scenes’ recommendation.

Normally, movie highlights mean the most exciting or memorable parts of a movie. However, the definition of exciting may vary for different kinds of movies. For example, the intensive motion segments in action movies tend to arouse the excitement of viewers, while in horror movies, even the abnormal still and quiet clips are very exciting for the audience. Apart from video content analysis, research problems of concern and methodologies in use for movie highlights’ extraction are also related to other technical fields, ranging from cognitive psychology [5,6] to cinematographic [4]. As a result, the problem of movie highlights’ extraction is rather challenging, simply because existing work in affective video analysis cannot be directly applied to it.

Of all the videos, as opposed to sports and news, movies are the most complex in structure and variability of content. Though it is relatively easy to predefine and determine certain events for specified videos, such as goal events in soccer, the disadvantage of event-based approaches is obvious. It is infeasible to take into account all highlight-related events for a movie in advance. As such, an unconventional model and approach beyond conventional event-based video analysis is required in this context. An early attempt by Hanjalic [19] used a group of local features to derive the high-level affective curve in emotion space for highlights’ extraction in sports video. Then, Reference [2] introduced their connotative properties and connotative space for affective description and classification.

When the human cognitive model is absent, there is a huge gap in conventional video content analysis for direct mappings from low-level features to high-level affective meanings. In this paper, a cognitive psychology-based perceptual model is proposed to bridge this gap. With huge data collected from a group of well-known movies, the model is applied for highlights’ extraction and perceptual analysis and understanding of movie videos. The realistic psychological reaction of the audience is obtained in a user survey. It is worth noting that the expected excitement curve of a movie should not be confused with the actual excitement response from individuals. While the latter is highly subjective and context dependent, the former is relatively objective, as it represents the average response of a movie audience. With experiments conducted on thousands of movie segments, the proposed model demonstrated how highlights are successfully extracted from movies, whilst the gap between low-level features and the high-level perceptual space of excitement is reduced.

The remaining part of this paper is organized as follows. Section 2 introduces our motivations and contributions. Section 3 presents the overall methodology in implementing the proposed model, including middle-level feature extraction, excitement time curve determination, and revealing the expected excitement level of the audience to the stimulus of the audio-visual effects’ variation. Experimental results and analysis are discussed in Section 4, with some concluding remarks drawn in Section 5.

## 2. Our Motivations and Contributions

Based on the description above, a prototype-based feature is proposed to measure how a specific event is different from its normal level. With this feature, the proposed approach can find out not only events with obvious high feature values, as done in Reference [31], but also segment with highly abnormal feature values. As the extraction of the excitement level in a video is relative to cognitive psychology, existing models need to be psychologically justifiable. Our work is inspired by the following three main principles:

- The cognitive psychology research framework should produce more reasonable results.
- In the bottom-up processing within the framework, a simple prototype approach-based local feature should be adopted instead of superposition of the features’ values.
- To enable top-down processing, the global expectation and sensitivity of the film should be estimated.

Based on the three principles above, the main contributions of this paper can be summarized as below and are detailed in the next section.

- A hybrid cognitive psychology model in combing bottom-up and top-down processing is proposed to mimic the excitement time curve of a film;
- A set of new global and local features measures the average sensitivity and abnormality of low-level features;
- A sensitivity adaptive algorithm to extract the excitement time curve of a film.

## 3. Overall Methodology

**Figure 1:**
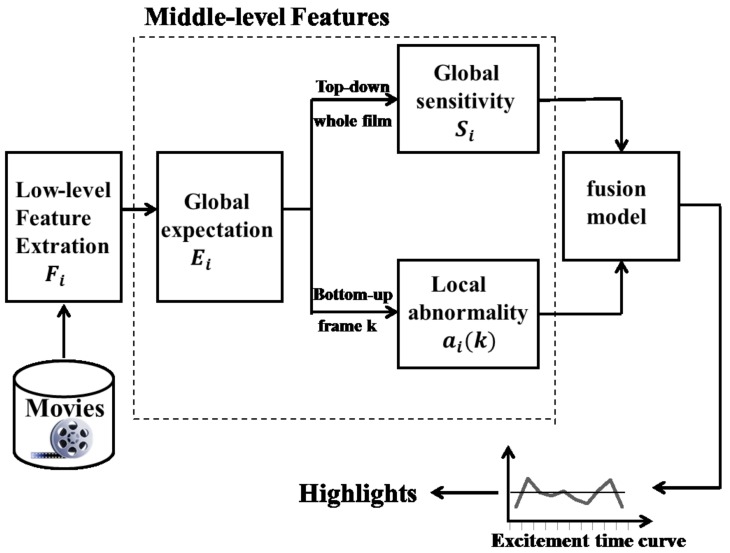
Proposed cognitive psychology framework-based model.

For movie highlights’ extraction, modeling of the excitement time curve is emphasized to fulfill the task in deriving global sensitivity and local abnormality values from the values of low-level features computed in a film [31]. Figure 1 illustrates the cognitive psychology-based framework for the implementation of the proposed automatic highlights’ extraction model. Firstly, low-level audio-visual features are extracted from a film, from which the global and local middle-level features are deduced for both top-down and bottom-up processing. Finally these middle-level features are fused together to obtain the excitement time curve and determine highlights by using a cutoff line. The criteria for model development and the proposed middle-level features are introduced in detail as follows.

### 3.1. Criteria for Developing Middle-Level Features

As excitement is a concept in psychology categories, its features need to be psychologically justifiable. Two criteria to reflect its correspondence to the psychology of people watching a film are introduced below.

The first criterion, global attribute, ensures that the values of some middle-level features obtained in the film are global descriptions for the whole film. This criterion projects our psychological characteristics before watching a film. The second criterion, local abnormality, accounts for the degree of how different the events are from their normal situation. It enables that not only the drastic events (such as fighting, explosions, screaming, etc.), but also the suspenseful ones, even with no obvious high values of low-level features, can be detected. The two criteria actually correspond to the top-down and bottom-up processing in the proposed framework.

### 3.2. Feature Selection

To date, many specific audio-visual features have been proposed to map videos to emotional dimensions of the audience. The most popular cues are motion intensity, cut density, and sound energy [19]. Although these three features reflect various patterns of many film grammars employed by directors to emphasize their work emotionally, new features are desirable to obtain more sophisticated results. In recent work, the facial expression of actors in a film was used to determine the emotional categories [40]. Other useful low-level features include color energy, lighting key, etc. [32,41,42], and deep learning methods [43,44,45,46]. However, these audio-visual features are still insufficient to deal with various patterns in a film, and their stabilities and universalities have not been thoroughly validated. Although only three basic features are used in this paper, the main contribution is that we probe new middle-level features in the field of cognitive psychology instead of testing new low-level audio-visual features. Nevertheless, exploring new features for the proposed framework is still an open option for future investigation.

As shown in Figure 1, the top-down processing in the framework is a composite of global expectation and global sensitivity. These two global features may bear relations to the psychology of the audience before they watch a film. The reason we propose these global features is that many researchers tend to use low-level features to map the high-level emotional dimension. However, these approaches are data-driven or bottom-up processing, which ignores the global expectation and sensitivity level of the audience to the film. For instance, the audience will normally be more expectant of and sensitive to the motion visual effects than other cues in an action movie. Another case, normally when we are watching a horror film, the sound effects should arouse in us more terrible feelings than other types of movies. On the contrary, in most dramas, neither the motion cues, nor the sound effects are more expected by and sensitive for the audience than the plot.

Another middle-level feature proposed in this paper is local abnormality, as shown in the bottom-up processing of Figure 1. This feature measures the degree of deviation between the low-level features’ values and their normal levels. The prototype method of the cognitive psychology field is the theoretical basis of this idea, and the motivation for which we propose this feature is that: as described in the previous sections, it is still a very difficult problem to extract the exact excitement time curve for the various editing schemes of a video.

**Figure 2:**
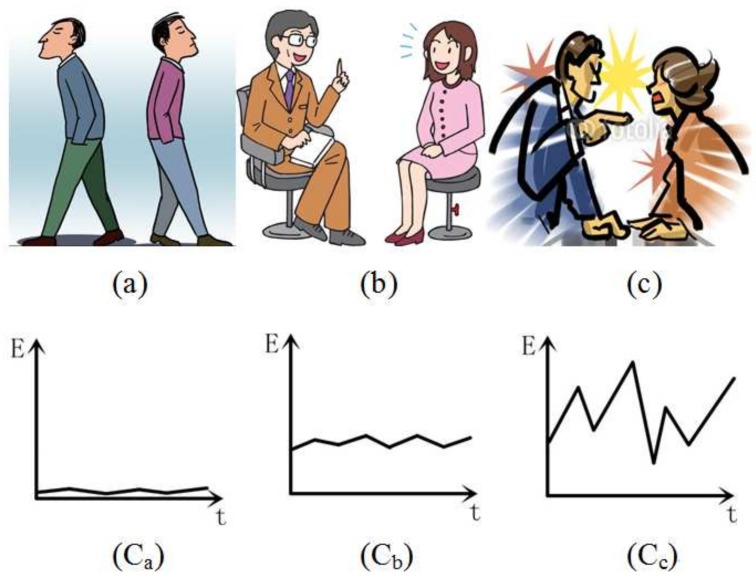
Different situations of people communicating and their probable sound feature curves, which include unconcerned behavior (**a**), normal talking (**b**), and quarreling (**c**).

As early work has argued, the expected variations in a user’s excitement included in the video can be modeled as a function of various content-unrelated video features [19,31]. However, these reported literature works assume that the excitement level is proportional to the values of motion, cut rate, and sound energy. Though this assumption works well in sports videos, this is not always the case in movies. For examples, a hero is holding his breath and hiding himself from an enemy, who is already very close to him. There is no obvious motion or sound effects in this scene, but the audience may still feel very excited. We have no interest in the way of detecting specific event patterns, but there is still a useful cue that an exciting event can be regarded as an abnormal situation compared to the normal one. To understand this definition, an example is shown in Figure 2. All three pictures show talking events, but Figure 2b, which illustrates two people talking normally, can be regarded as a normal situation or a prototype, and Figure 2a,c, which describes estranged and quarrel situations, are abnormal situations. As shown in Figure 2, the sound energy values of situations in Figure 2a–c reveal a more visible difference between these events. The deviation of these events’ low-level feature is called the local abnormality feature in this paper and is adopted as the local middle-level feature to calculate the affective dimension.

### 3.3. Model for the Excitement Time Curve

As described in Reference [31], the proposed method starts by considering the function $G_{i}\left(k\right)$, which models the changes in the excitement dimension over the frame *K* as revealed by the feature *i*. The expected variations in a user’s excitement H(k) while watching a video should be composed of *N* basic components $G_{i}\left(k\right)$, as formulated in Equation (1).

(1) $$H\left(k\right)=F(G_{i}\left(k\right),i=1,\ldots ,N)$$

However, as described in previous sections, this formulation presents a simple bottom-up processing, which does not take the global user’s psychology factors into account. Moreover, the above model considers only the situation that the excitement level is proportional to the features’ value, which only works in sport videos, but is not always the case in movies.

Let the parameters $E_{i}$ and $S_{i}$ be the global features of the average user’s expectation of and sensitivity to feature *i*, respectively. Consider the function $D_{i}\left(k\right)$ that models the changes in the deviation of feature *i* to its corresponding expectation (or prototype) over the frame *K* in a movie. Then, we model the excitement time curve H(k) in general as a function of *N* components $D_{i}\left(k\right)$ and a set of global parameters in Equation (2).

(2) $$H\left(k\right)=\tilde{F}\left(E_{i},S_{i},D_{i}\left(k\right),i=1,\ldots ,N\right)$$

Here, the function $\tilde{F}$ serves to select different weights to integrate the contributions of all components $D_{i}\left(k\right)$ in the whole processing of calculating H(k) along a video according to $E_{i}$ and $S_{i}$. Then, we will elaborate how to search for the appropriate form of the function Equation (2) and evaluate it on a number of test sequences.

#### 3.3.1. Low-Level Features

Function $D_{i}\left(k\right)$ is based on components $G_{i}\left(k\right)$, so we first introduce the selection of low-level features. Although we think that in the proposed model, any low-level feature can be selected, which represents the stimuli that will likely influence the affective state of a user while watching a movie, only the psychophysiological experiments’ validated low-level features are adopted in this paper. They are sound energy e(k), motion intensity m(k), and shot cut rate c(k). These features are expected to cause the change of the excitement level of the user while watching a movie. We use the same method in Reference [31] to extract their values.

**Figure 3:**
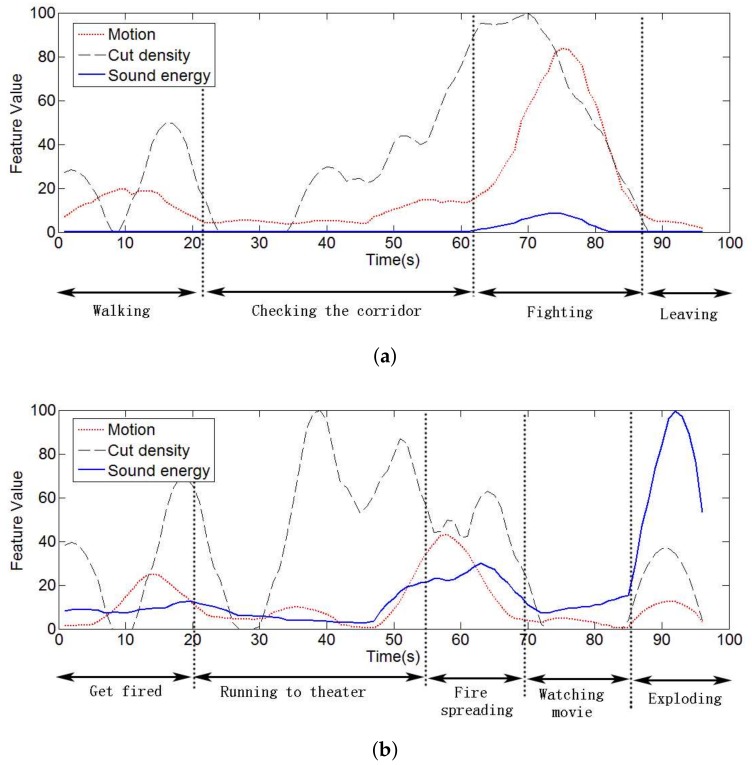
Low-level feature time curves of two short segments.

To test the relation between the low-level features and specific events, we extracted the features’ time curves from two segments of famous films, and each curve was filtered by a Kaiser window for the smooth effects, as shown in Figure 3.

As labeled in Figure 3a, this scene consists of four events, including people walking, people checking the corridor, people fighting, and people leaving. Among these events, fighting is more attractive to users for the drastic action and frequent shot cutting. Another example of movie scenes as shown in Figure 3b has more complex events. This scene describes a story in which the hero runs to the theater to stop a potential explosion, but fails ultimately. The close-up of fire in the event of fire spreading caused a high level of motion intensity level. At the end of the segment, the explosion event accompanies a huge volume of sound.

Two conclusions can be drawn from the test: (a) there is definitely a relation between the low-level features and high-level excitement dimension; (b) different movies involve different sensitivities in the types of low-level features’ values, so more flexible middle-level features are needed.

#### 3.3.2. Global Expectation and Sensitivity Features

People subconsciously show different expectations and sensitivities to different types of audio-visual features in watching different types of movies [10]. For examples, in action movies, the motion strength tends to be more stimulating than other effects. In horror movies, sound effects play an important role in the user’s emotion. We aim at obtaining global features of the whole movie that reveal the sensitivity between the user’s excitement level and different features. We start modeling the sensitivity by defining the global expectation feature $E_{i}$ responding to feature *i*.

(3) $$E_{i}=\frac{\sum_{k=1}^{n}G_{i}\left(k\right)}{n},i=1,\ldots ,N$$

Here, *n* is the total frame number of a movie. This feature is the average value of the feature in a movie. We also regard it as the specific feature value of a normal event or a prototype. Then, we calculate two variances in Equations (4) and (5).

(4) $$V_{i}=\frac{{\left(G_{i}\left(k\right)-E_{i}\right)}^{2}}{n}$$

(5) $$V_{i}^{\prime }=\frac{{\left(G_{i}\left(k^{\prime }\right)-E_{i}\right)}^{2}}{n^{\prime }}$$

Here, $k^{\prime }$ is the frame index where $G_{i}\left(k^{\prime }\right)>E_{i}$ and $n^{\prime }$ is the total number of $k^{\prime }$. $V_{i}$ represents the variance of feature *i* in a film, and $V_{i}^{\prime }$ represents the variance of feature *i*, whose value is larger than $E_{i}$. Then, we define the global sensitivity feature $S_{i}$ in the Formulation (7).

(6) $$R_{i}=\frac{V{}_{i}^{\prime }}{V_{i}},i=1,\ldots ,N$$

(7) $$S_{i}=\left\{\begin{array}{c} R_{i},\;ifR_{i}\leq 1.0\\ 1.0,\;ifR_{i}>1.0 \end{array}\right.$$

The feature $S_{i}$ represents the activity level of feature *i* in a movie. Its maximum value is 1.0, which means that this feature is high in activity in the film and that the audience should be most sensitive to it.

#### 3.3.3. Local Abnormality Feature

In Section 3, we introduce the prototype method in the cognitive psychology research framework. Inspired by a similar idea, we propose a local abnormality feature to measure how far a feature deviates from the prototype while the feature changes along the film. Consider the local abnormality feature $A_{i}\left(k\right)$ over the frame *K* as revealed by the feature *i* defined as:

(8) $$A_{i}\left(k\right)=\left|G_{i}\left(k\right)-E_{i}\right|$$

**Figure 4:**
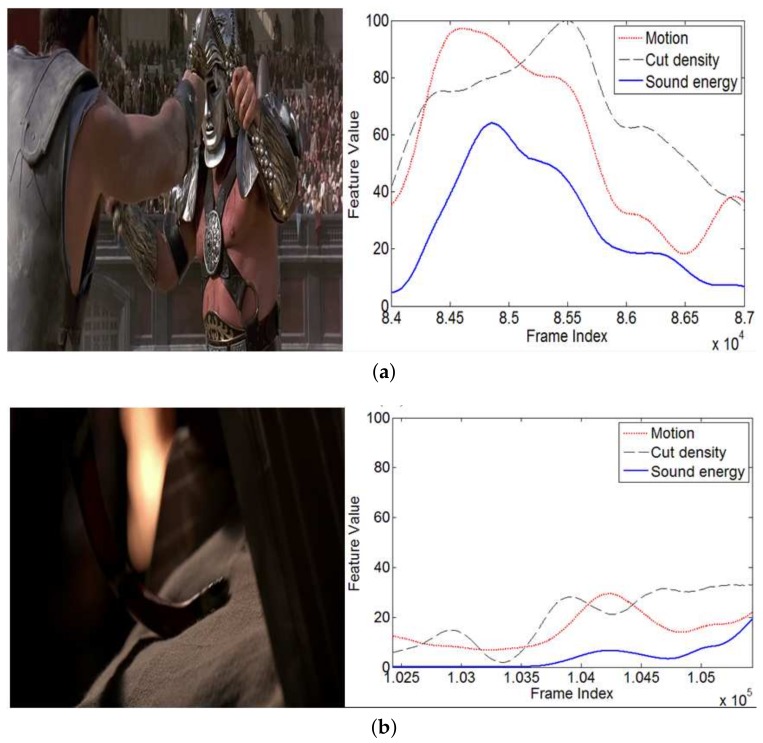
Examples of two exciting events with different feature curves. The low-level feature curves (right column) are extracted from two minutes of neighbor segments, which are centered on the frames corresponding to the screenshots on the right, respectively. (**a**) Two gladiators are fighting; (**b**) the enemy is using a poisonous snake to murder targets.

Here, the global expectation $E_{i}$ is regarded as the prototype of feature *i*. The absolute value of a difference value is used here, because we consider that the deviation degree of the audio-video effects accounts for the rising emotion level of the user. This is different from References [19,31], where the excitement level was always in a direct proportion to the feature values. For example, in an action movie, a hero may defeat his enemy by a violent attack. On the contrary, he may assassinate an enemy quietly and slowly. Both of the events will evoke the audience’s excitement emotion. However, two cases show completely different audio-visual feature values. Figure 4 shows examples of these situations. The picture is captured from the film “Gladiator”.

#### 3.3.4. Excitement Model

The excitement time curve H(k) is fused by multiple stimuli represented by the audio-visual feature time curve $G_{i}\left(k\right)$. To obtain a more accurate result, the proposed cognitive framework and middle-level features will be taken into account. For top-down processing, the global sensitivity feature will affect the global effect of a low-level feature; for bottom-up processing, the local abnormality feature measures the deviation degree between an event and the prototype. The whole computing process is described as follows:

1. Considering the time stamp *k*, compute the values of the components $G_{i}\left(k\right)$ computed at that time stamp;
2. Calculate global expectation $E_{i}$ and sensitivity $S_{i}$ as in Equations (3) and (7);
3. Compute the local abnormality $A_{i}\left(k\right)$, as in Equation (8);
4. Using the fusion model to obtain a excitement time curve, the model is defined as follows
  (9) $$G_{i}^{\prime }\left(k\right)=G_{i}\left(k\right)w_{i}\left(k\right),i=1,\ldots ,N$$
  where:
  (10) $$w_{i}\left(k\right)=\frac{s_{i}}{2}\left(1+erf\left(\frac{A_{i}\left(k\right)}{\sigma }\right)\right)$$
  and:
  (11) $$erf\left(x\right)=\frac{2}{\sqrt{\pi }}\int_{0}^{x}e^{-t^{2}}dt$$
  The parameter σ is the spread factor determining the steepness of the curve. The weighting function Equation (10) ensures that any event that is abnormal and whose audio-visual effects are very sensitive for the user will be detected. On the contrary, normal events will be ignored.
5. After obtaining the filtered time curves $G_{i}^{\prime }\left(k\right)$, we fuse them as follows to obtain the excitement time curve H(k).
  (12) $$H\left(k\right)=\sum_{i}\eta_{i}G_{i}^{\prime }\left(k\right)$$
  Parameters $\eta_{i}$ are weighting factors of fusion $G_{i}^{\prime }\left(k\right)$ with $\sum_{i}\eta_{i}=1$.
6. To make the curve smooth, the excitement time curve H(k) is filtered by a Kaiser window k(ι,β), as shown in the following.
  (13) $$\tilde{H}\left(k\right)=k\left(\iota ,\beta \right)\times H\left(k\right)$$
7. By applying a cutoff line to the excitement time curve $\tilde{H}\left(k\right)$, only those segments whose values are higher than the value of cutoff line will be extracted as highlights.

## 4. Experiment

### 4.1. Data Collection and Experiment Setup

**Figure 5:**
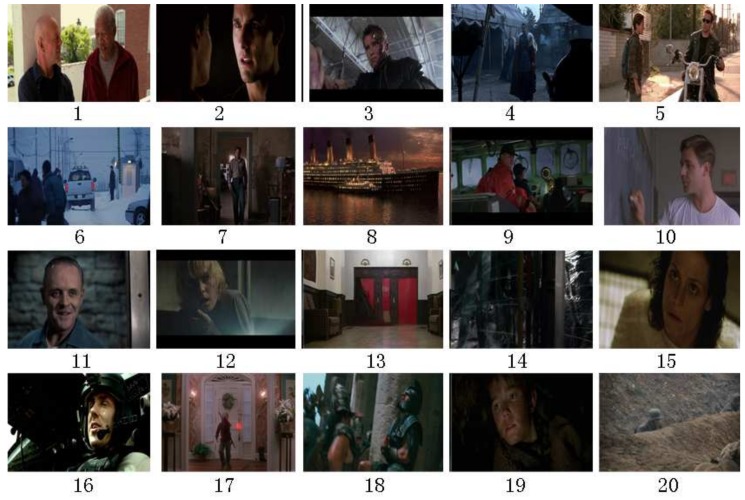
Key-frames from the testing database.

**Figure 6:**
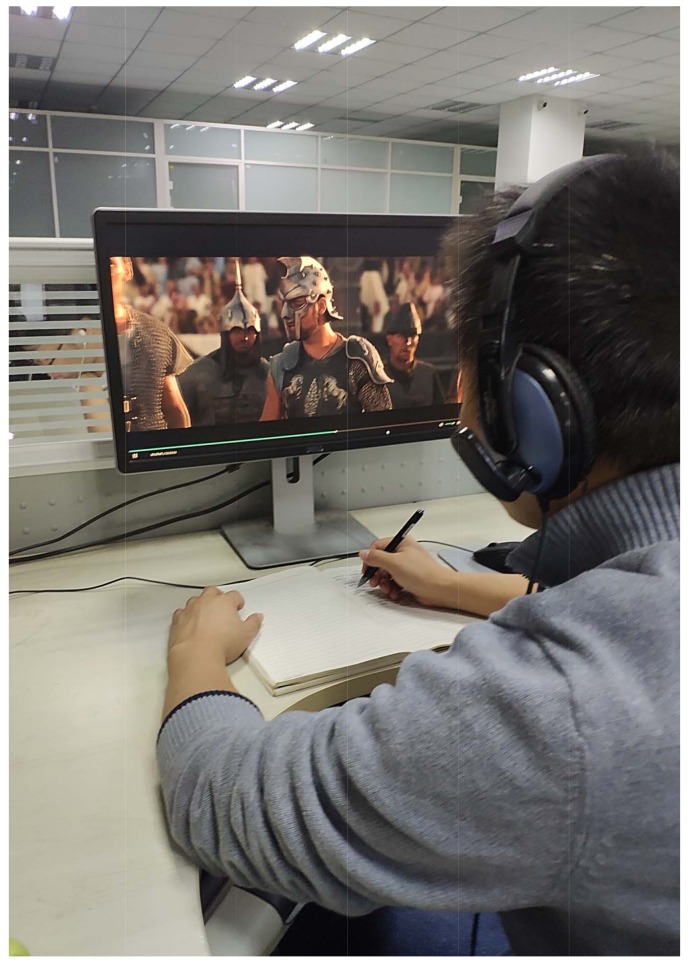
The process of dataset collection.

In our movie highlights’ extraction testing dataset, in total, 20 famous movies were selected for testing, which covers several major movie genres including action, horror, war, disaster, drama, etc. Examples of the screenshot of each movie are shown in Figure 5. In order to extract the highlights in the dataset of the 20 movies, we invited a number of testers to participate in the project. Each testee manually recorded the highlight time point when viewing the movies clips. Figure 6 shows the process of data collection.

**Figure 7:**
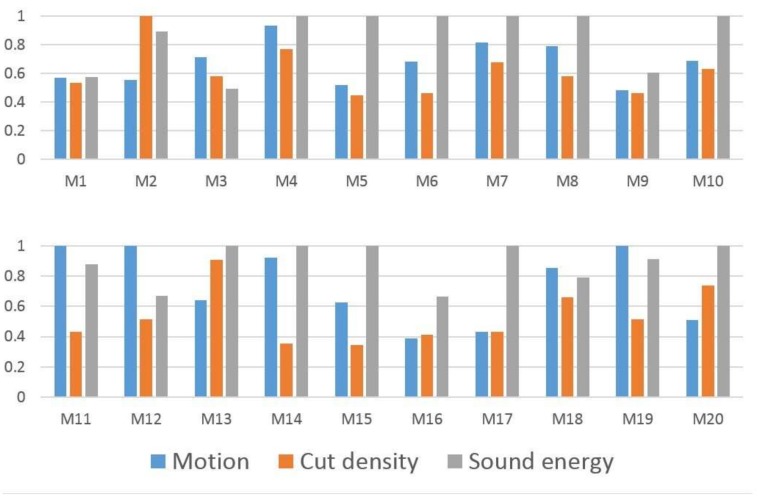
The global sensitivity feature values of 20 movies.

The global sensitivities’ results are shown in Figure 7. It can be seen that most movies are very sensitive with respect to sound effect. This has also been corroborated in Reference [32], as audio cues are often more informative than visual ones. On the other hand, motion cues are normally more sensitive than cut density, because motion cues carry more information to indicate content changes and the excitement of movie clips, even with limited cuts contained.

### 4.2. Case Study

In order to evaluate the performance of the proposed method, we extracted the excitement time curve on all 20 movies and studied them according to a group of predefined events within these films. For benchmarking purposes, our results were comparatively analyzed with Hanjalic’s algorithm [19]. A detailed discussion on three famous movies, “Gladiator (2000)”, “Saving Private Ryan (1998)”, and “The Shining (1997)”, are presented below; these were selected as they are well-known and reflect a high diversity of genres of movies.

**Figure 8:**
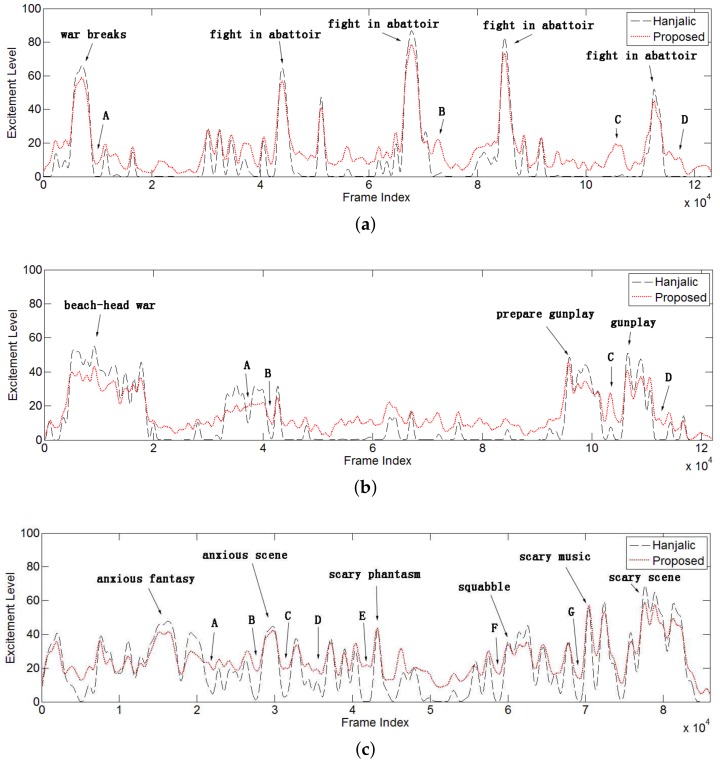
Excitement time curves extracted from three films including “Gladiator” (**a**), “Saving Private Ryan” (**b**), and “The Shining” (**c**).

For each of the three selected movies, the excitement time curve is extracted and plotted in Figure 8. By specifying the predefined events in these excitement time curves, the corresponding excitement levels associated with such events were identified. Ideally, the excitement level for these events should be high to enable them to be included as highlights.

#### 4.2.1. Detecting Violent Events

**Figure 9:**
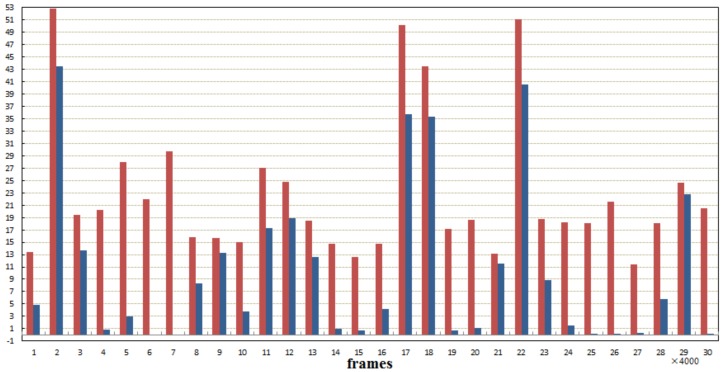
Comparison results with unsupervised extraction of video highlights via robust recurrent auto-encoders [27]. The red columns are the highlight values calculated by using the proposed method, and the blue columns are the results of [27]. It can be noticed that the proposed method can find more highlight segments.

Violent events contain higher feature values with a higher weight factor for fusion processing, which forms obvious peaks on the curve. In Figure 9, most peaks are labeled with corresponding events manually. It can be concluded from the results that our method performed at the same level as Hanjalic’s to detect the violent events like fighting in the abattoir, gunplay, some horrible scenes, etc.

#### 4.2.2. Detecting Exciting Events with Low Feature Values

Though both the proposed approach and Hanjalic’s [19] yielded consistent high peaks in excitement curves for violent event detection, there were some notable differences in the curves at the same frame index. For clarity, these positions are labeled with capital letters in Figure 8, corresponding to several key events in the three movies. These events are summarized in Table 1, Table 2 and Table 3 along with the description and the emotional feeling of the event.

Actually, all these events can provoke various kinds of excitement. The difference above indicated that the proposed combined cognitive psychology model outperformed [19] in extracting these events even with insignificant audio-visual effects. Additionally, sudden slow motion and unusual silent effects are adopted by movie directors to reflect by contrast the excitement level of certain events. In these cases, our proposed approach performed particularly better at generating consistent and continuous excitement curves. Examples can be found for the sampled event points in Figure 8, including Event A for “Gladiator”, Events A–C for “Saving Private Ryan” and Event C for “The Shining”. In contrast, Hanjalic’s approach [19] had a near-zero response for these events.

All scenes in these tables have different types of emotional effects on the audience. Whatever the emotional types are, they can arouse our excitement level. Normally, these scenes do not show an obvious increase of the low-level features that were employed is this paper, which is the reason their excitement levels were very low in Hanjalic’s result. As for the proposed method, we could detect the abnormalities of the events, which led to the improvement of our results.

#### 4.2.3. Curve Continuity Discussion

During the process of some exciting events of movies, the director may employ some special effects to achieve more impactive effects on the audience’s emotion, like deep sound, slow motion, and low cut density. Normally, this segment is part of a whole exciting event. For example, at Position A of “Gladiator” shown in Figure 8, this is the end part of a horrifying war and presents several battle scenes with slow motion and deep background music; so are the A, B and C positions of “Saving Private Ryan” and C position of “The Shining”. To mimic the excitement level of a movie, the proposed method showed more continuity inside the curve of the whole event than Hanjalic’s, whose curve showed that the whole event curve was almost divided into two segments. Furthermore, our results contained almost no zero values. This is reasonable because film is elaborated with a compact story line. It is not possible that the audience’s excitement level could decrease to zero. Finally, it can be concluded that the experimental results demonstrate the effectiveness of the proposed method in extracting the excitement level of the audience.

#### 4.2.4. Comparison Result

Table 4 shows the results of the proposed method running on the eight movies. According to the results, it can be concluded that the proposed method significantly improved the accuracy of movie highlights’ detection, especially for horror movies.

## 5. Conclusions

In this paper, a two-way movie highlights model is proposed for more realistic simulation of the human perception of excitement levels. Combining with top-down and bottom-up processing, two criteria, global sensitivity and local abnormality are introduced for the development of middle-level features. According to various sensitivity levels of individual viewers to different low-level audio-visual features, the fused results can be adjusted in the prototype-based approach within our proposed model. The proposed approach generates an excitement time curve by the fusion of several low-level video features, each of which corresponds to the changes in the user’s excitement as a reaction to the stimulus of movies. It reduces the gap between low-level audio-visual features and high-level affective semantic analysis of movies.

## Figures and Tables

**Table 1:** Gladiator.

No.	Time Periods	Description	Affect
A	0:11:30–0:12:28	A very horrifying battle scene is shown with slow motion and deep music.	Horrifying
B	1:38:11–1:38:57	Hero is hiding an arrow head in his hand and wants to kill the evil king.	Tension
C	2:24:19–2:26:11	Enemy put a snake in a bed to assassinate target quietly.	Dangerous
D	2:38:07–2:42:58	When hero killed the evil king, all the audience turns to quiet suddenly, and the hero sees a vision of his family, then dies.	Happy, anxious, and very sad

**Table 2:** Saving Private Ryan.

No.	Time Periods	Description	Affect
A	0:51:04–0:53:03	Teammate is shot by sniper, and all soldiers are hiding without movement and sound.	Dangerous
B	0:55:10–0:56:20	Our sniper killed enemy the sniper covertly and quietly.	Tension
C	2:21:56–2:26:54	A soldier is too scared to speak and walk and cries when an enemy passes him.	Dangerous
D	2:35:12–2:37:15	Hero is dying and leaving his last words.	Sad

**Table 3:** The Shining.

No.	Time Periods	Description	Affect
A	0:28:32–0:30:22	The kid is riding a bike along an empty long corridor.	Strange
B	0:36:19–0:38:16	The kid sees a terrible phantom, and he cannot cry or run away.	Scared
C	0:42:58–0:44:15	The kid is playing in an empty hall alone; suddenly, a door is opened, but is nobody there.	Dangerous
D	0:48:32–0:51:45	The leading man dropped into the illusion that he is drinking in a bar, and he is talking to himself.	Strange
E	0:56:48–1:00:36	The leading man is kissing a beauty, but he catches a glimpse that she is a zombie.	Horrible
F	1:20:24–1:23:45	The leading man wants to get close to the leading lady quietly and kill her.	Dangerous
G	1:35:19–1:37:28	The kid is holding a dagger and standing beside the sleeping leading lady.	Horrible

**Table 4:** Compared results.

No.	Movie Names	GT	Our	Hanjalic
M1	Gladiator	15	13	11
M2	Titanic	12	10	9
M3	The Perfect Storm	17	13	13
M4	Silent Hill	18	16	10
M5	The Shining	16	14	9
M6	Troy	29	24	24
M7	Brave heart	20	18	16
M8	Saving Private Ryan	27	25	24

## References

[1] Taşdemir K., Cetin A.E. (2014). Content-based video copy detection based on motion vectors estimated using a lower frame rate. Signal Image Video Process..

[2] Chang S.F., Vetro A. (2005). Video adaptation: Concepts, technologies, and open issues. Proc. IEEE.

[3] Canini L., Benini S., Leonardi R. (2013). Affective recommendation of movies based on selected connotative features. IEEE Trans. Circuits Syst. Video Technol..

[4] Arijon D. (1976). Grammar of the Film Language.

[5] Darwin C., Prodger P. (1998). The Expression of the Emotions in Man and Animals.

[6] Arnold M.B. (1960). Emotion and Personality.

[7] Gaikwad R., Neve J.R. A comprehensive study in novel content based video retrieval using vector quantization over a diversity of color spaces. Proceedings of the International Conference on Global Trends in Signal Processing, Information Computing and Communication.

[8] Mittal A., Cheong L.F. (2003). Framework for synthesizing semantic-level indices. Multimed. Tools Appl..

[9] Yeh M.C., Tsai Y.W., Hsu H.C. (2016). A content-based approach for detecting highlights in action movies. Multimed. Syst..

[10] Irie G., Satou T., Kojima A., Yamasaki T., Aizawa K. (2010). Affective audio-visual words and latent topic driving model for realizing movie affective scene classification. IEEE Trans. Multimed..

[11] Zeng Z., Pantic M., Roisman G.I., Huang T.S. (2009). A survey of affect recognition methods: Audio, visual, and spontaneous expressions. IEEE Trans. Pattern Anal. Mach. Intell..

[12] Poria S., Peng H., Hussain A., Howard N., Cambria E. (2017). Ensemble application of convolutional neural networks and multiple kernel learning for multimodal sentiment analysis. Neurocomputing.

[13] Paul S., Saoda N., Rahman S.M.M., Hatzinakos D. Mutual information-based selection of audiovisual affective features to predict instantaneous emotional state. Proceedings of the International Conference on Computer and Information Technology.

[14] Zhang S., Zhang S., Huang T., Gao W., Tian Q. (2018). Learning Affective Features with a Hybrid Deep Model for Audio-Visual Emotion Recognition. IEEE Trans. Circuits Syst. Video Technol..

[15] Omidyeganeh M., Ghaemmaghami S., Shirmohammadi S. (2013). Group-based spatio-temporal video analysis and abstraction using wavelet parameters. Signal Image Video Process..

[16] Xu M., Chia L.T., Jin J. Affective content analysis in comedy and horror videos by audio emotional event detection. Proceedings of the IEEE International Conference on Multimedia and Expo.

[17] Zhalehpour S., Akhtar Z., Erdem C.E. (2016). Multimodal emotion recognition based on peak frame selection from video. Signal Image Video Process..

[18] Xu M., Wang J., He X., Jin J.S., Luo S., Lu H. (2014). A three-level framework for affective content analysis and its case studies. Multimed. Tools Appl..

[19] Hanjalic A. (2005). Adaptive extraction of highlights from a sport video based on excitement modeling. IEEE Trans. Multimed..

[20] Hu W., Xie N., Li L., Zeng X., Maybank S. (2011). A survey on visual content-based video indexing and retrieval. IEEE Trans. Syst. Man Cybern. Part C (Appl. Rev.).

[21] Geetha P., Narayanan V. (2008). A Survey of Content-Based Video Retrieval. J. Comput. Sci..

[22] Del Bimbo A. (1999). Visual Information Retrieval.

[23] Hanjalic A., Langelaar G., Van Roosmalen P., Biemond J., Lagendijk R. (2000). Image and Video Databases: Restoration, Watermarking and Retrieval.

[24] Lew M.S. (2013). Principles of Visual Information Retrieval.

[25] Feng W., Jia J., Liu Z. (2010). Self-Validated Labeling of Markov Random Fields for Image Segmentation. IEEE Trans. Pattern Anal. Mach. Intell..

[26] Wang S., Ji Q. (2015). Video Affective Content Analysis: A Survey of State-of-the-Art Methods. IEEE Trans. Affect. Comput..

[27] Yang H., Wang B., Lin S., Wipf D., Guo M., Guo B. Unsupervised Extraction of Video Highlights via Robust Recurrent Auto-Encoders. Proceedings of the 2015 IEEE International Conference on Computer Vision (ICCV).

[28] Xu Y., Han Y., Hong R., Tian Q. (2018). Sequential Video VLAD: Training the Aggregation Locally and Temporally. IEEE Trans. Image Process..

[29] Zhao S., Yao H., Sun X. Video classification and recommendation based on affective analysis of viewers. Proceedings of the ACM International Conference on Multimedia.

[30] Zhao S., Yao H., Sun X., Jiang X., Xu P. Flexible Presentation of Videos Based on Affective Content Analysis. Proceedings of the International Conference on Multimedia Modeling.

[31] Hanjalic A., Xu L.Q. (2005). Affective video content representation and modeling. IEEE Trans. Multimed..

[32] Wang H.L., Cheong L.F. (2006). Affective understanding in film. IEEE Trans. Circuits Syst. Video Technol..

[33] Kang H.B. Affective content detection using HMMs. Proceedings of the Eleventh ACM International Conference on Multimedia.

[34] Rasheed Z., Sheikh Y., Shah M. (2005). On the use of computable features for film classification. IEEE Trans. Circuits Syst. Video Technol..

[35] Zhang S., Huang Q., Jiang S., Gao W., Tian Q. (2010). Affective visualization and retrieval for music video. IEEE Trans. Multimed..

[36] Zhu G., Xu C., Huang Q., Rui Y., Jiang S., Gao W., Yao H. (2009). Event tactic analysis based on broadcast sports video. IEEE Trans. Multimed..

[37] Krippendorff K. (2012). Content Analysis: An Introduction to Its Methodology.

[38] Xu G., Ma Y.F., Zhang H.J., Yang S. A HMM based semantic analysis framework for sports game event detection. Proceedings of the 2003 International Conference on Image Processing.

[39] Xu M., Jin J.S., Luo S., Duan L. Hierarchical movie affective content analysis based on arousal and valence features. Proceedings of the 16th ACM international conference on Multimedia.

[40] Lin J.C., Wu C.H., Wei W.L. (2012). Error weighted semi-coupled hidden Markov model for audio-visual emotion recognition. IEEE Trans. Multimed..

[41] Rui H., Wei F., Jizhou S. (2017). Color feature reinforcement for co-saliency detection without single saliency residuals. IEEE Signal Process. Lett..

[42] Qing G., Sun S., Ren X., Dong F., Gao B., Feng W. (2018). Frequency-tuned active contour model. Neurocomputing.

[43] Han J., Cheng G., Li Z., Zhang D. (2018). A Unified Metric Learning-Based Framework for Co-saliency Detection. IEEE Trans. Circuits Syst. Video Technol..

[44] Han J., Chen H., Liu N., Yan C., Li X. (2018). CNNs-Based RGB-D Saliency Detection via Cross-View Transfer and Multiview Fusion. IEEE Trans. Cybern..

[45] Cheng G., Han J., Zhou P., Xu D. (2018). Learning Rotation-Invariant and Fisher Discriminative Convolutional Neural Networks for Object Detection. IEEE Trans. Image Process..

[46] Zhang D., Han J., Zhao L., Meng D. (2018). Leveraging Prior-Knowledge for Weakly Supervised Object Detection under a Collaborative Self-Paced Curriculum Learning Framework. Int. J. Comput. Vis..

